# Effect of the Degenerative State of the Intervertebral Disk on the Impact Characteristics of Human Spine Segments

**DOI:** 10.3389/fbioe.2013.00016

**Published:** 2013-12-16

**Authors:** Sara E. Wilson, Ron N. Alkalay, Elizabeth Myers

**Affiliations:** ^1^Department of Mechanical Engineering, University of Kansas, Lawrence, KS, USA; ^2^Department of Orthopedics, Center for Advanced Orthopedic Studies, Beth Israel Deaconess Medical Center, Harvard Medical School, Boston, MA, USA; ^3^Doris Duke Charitable Foundation, New York, NY, USA

**Keywords:** intervertebral disk, experimental study, dynamic characteristics, magnetic resonance imaging, non-linear stiffness, vertebral fracture, falls

## Abstract

Models of the dynamic response of the lumbar spine have been used to examine vertebral fractures (VFx) during falls and whole body vibration transmission in the occupational setting. Although understanding the viscoelastic stiffness or damping characteristics of the lumbar spine are necessary for modeling the dynamics of the spine, little is known about the effect of intervertebral disk degeneration on these characteristics at high loading rates. We hypothesize that disk degeneration significantly affects the viscoelastic response of spinal segments to high loading rate. We additionally hypothesize the lumbar spine stiffness and damping characteristics are a function of the degree of preload. A custom, pendulum impact tester was used to impact 19 L1–L3 human spine segments with an end mass of 20.9 kg under increasing preloads with the resulting force response measured. A Kelvin–Voigt model, fitted to the frequency and decay response of the post-impact oscillations was used to compute stiffness and damping constants. The spine segments exhibited a second-order, under-damped response with stiffness and damping values of 17.9–754.5 kN/m and 133.6–905.3 Ns/m respectively. Regression models demonstrated that stiffness, but not damping, significantly correlated with preload (*p* < 0.001). Degenerative disk disease, reflected as reduction in magnetic resonance T2 relaxation time, was weakly correlated with change in stiffness at low preloads. This study highlights the need to incorporate the observed non-linear increase in stiffness of the spine under high loading rates in dynamic models of spine investigating the effects of a fall on VFx and those investigating the response of the spine to vibration.

## Introduction

Vertebral fractures are the second most common fracture site for individuals with osteoporosis (Melton et al., 1989; Cooper et al., 1992; Anonymous, 1998; Johnell and Kanis, 2005; Taylor et al., 2011) with a high proportion (30–50%) of patients reporting a fall event preceding the occurrence of spinal fracture (Cooper et al., 1992; Myers and Wilson, 1997). In older men, vertebral fractures (VFx) in the lumbosacral spine represented 56% of all reported fracture cases (Freitas et al., 2008). In 2001, hospital emergency departments treated an estimated 1.64 million elderly adults for accidental falls in hospital emergency departments in the USA (Stevens and Sogolow, 2005), with number of patients increasing by 10.5% between 2001 and 2008 when adjusted for relative growth of this age population during this period. Spinal cord injuries were associated with 8 and 26% of falls from standing height or lower (O’Connor, 2002; Freitas et al., 2008). Associated pain and the considerable physical and the degree of psychological/discomfort lead to functional impairments in up to 30% of these patients (Cooper et al., 1992; Lyles et al., 1993; Gold, 1996). Dynamic modeling of a backwards fall demonstrates that the resultant impact can propagate up the lumbar spine producing in forces that exceed the failure load of elderly cadaveric vertebrae (Wilson and Myers, 1998; Wilson, 1999). These models, however, are highly dependent on accurate modeling of the spine stiffness and damping characteristics.

Similarly, whole body vibration has been identified as one of five major risk factors for low back pain and modeling of whole body vibration has been used to better understand the propagations of vibration from seat to spine and to assess the possible mechanisms for spine injury due to vibration (Bernard, 1997; Boileaua É and Rakhejab, 1998). These models also require accurate models of high-rate, spine, viscoelastic characteristics.

While several studies have examined stiffness and damping characteristics of spine segments, many of these have examined stiffness characteristics at slower rates of loading and have not examined the effects of disk degeneration of high-rate stiffness characteristics. Within the structure of the functional spinal unit (FSU), the intervertebral disk joint confers mobility and provides static and dynamic load attenuation to the spinal column (Brown et al., 1957; Bodine et al., 1982; Adams and Hutton, 1983; Brinckmann et al., 1983; Burns et al., 1984; Adams et al., 1994; Botsford et al., 1994; Adams et al., 1996) with a viscoelastic response exhibiting time constants on the order of hours (Keller and Spengler, 1987). Kasra et al. (1992) reported peak transmission for human thoraco-lumbar specimens to occur at frequencies of between 23.5 and 33 Hz for a mass of 40 kg with the FSU showing damping ratios of 0.05 and 0.13. This corresponds to a single FSU stiffness of 872–1720 kN/m and damping constant of 590–2156 Ns/m. Employing six lumbar FSU specimens, Rostedt et al. (1998) found stiffness values to range between 1400 and 2800 kN/m with the stiffness increasing with preload. These values are much higher than the instantaneous stiffness found in creep studies (Keller and Spengler, 1987), suggesting the rate of loading to be an important contributor to FSU stiffness. Degenerative changes in the disk’s annulus and nucleus, which strongly affect their static and viscoelastic material properties (Acaroglu et al., 1995; Ebara et al., 1996; Iatridis et al., 1998) and the associated loss of hydration in the disk’s tissues (Galante, 1967; Panagiotacopulos et al., 1987; Best et al., 1994), result in the degradation of the FSU’s long-term creep behavior and energy dissipation (Lai et al., 1991; Laible et al., 1993; Best et al., 1994). Although the degenerative state of the disk and its hydration state are important determinants of the spine’s stiffness and damping characteristics, little is known about their role in affecting high-rate dynamics of spine segments. Consequently, little is also known about the role of disk degeneration in the vertebra failure of the elderly spine when exposed to high-rate loading associated with a fall event.

In view of the obvious limitations on *in vivo* investigation of VFx with falls, experimental (Moro et al., 1995; Myers and Wilson, 1997; Rostedt et al., 1998; Ochia and Ching, 2002) and computational studies (Wilson and Myers, 1998; Wilson, 1999; Van Toen et al., 2012) were conducted to define a VFx risk, defined as a ratio of the force resulting in the failure of the vertebra under a set activity vs. the peak force applied (Myers and Wilson, 1997). Though several previous studies have investigated the response of the spine to dynamic loading (Kong and Goel, 2003; Guo and Teo, 2005; Guo et al., 2009), high-rate loading (Belytschko and Privitzer, 1978; Belytschko et al., 1978; Luo and Goldsmith, 1991), and force on the buttocks in subinjurious fall (Sran and Robinovitch, 2008; Van Toen et al., 2012), these models pertain mostly to the young to middle age, male population that might be subject to workplace injuries. Wilson and Myers (1998), employing an optimization based geometric model of osteopenic thoraco-lumbar spine, predicted the stiffness and damping characteristics of the spinal segments to influence the peak forces sustained by the vertebrae due to a backwards fall from standing height. For example, a 10% increase in the stiffness of the segment was predicted to yield an increase of 2–5% in the peak forces acting on the vertebrae (Wilson and Myers, 1998). This increase may predispose osteoporotic individuals to an increased risk of VFx, suggesting the assessment of the disk’s dynamic properties to be an important factor in quantifying VFx due to a backwards fall in the elderly.

Magnetic resonance imaging (MRI), the diagnostic tool of choice for clinical evaluation of spinal pathology (Boos et al., 1993; Boos and Boesch, 1995; Cassar-Pullicino, 1998; Haughton, 2004), relies mainly on assessing the loss of anatomical features and associated hydration, measured as a loss in signal intensity on T2-weighted images, within the disk (Tertti et al., 1991; Boos et al., 1993; Boss et al., 1993). T1 and T2 relaxation rates show an inverse relationship with the disk’s water content (Crooks et al., 1987; Fullerton and Camron, 1988) and its glycosaminoglycan content (Boos and Boesch, 1995). In view of the intimate relationship between the disk’s hydration and its mechanical competence, we hypothesize that MR classification of degenerative changes will be associated with the loss of dynamic stiffness and damping properties of the spinal segments. Furthermore, as Rostedt et al. (1998) observed stiffness is a function of preload, we also hypothesize that measured dynamic stiffness and damping properties will increase with preload applied to the spine.

## Materials and Methods

### Specimens and preparation

Five male and 14 female, L1–L3 cadaveric spine segments were obtained fresh-frozen from donors aged 62–85 years of age (mean age 75.5 ± 6.9 years) through the Harvard Anatomic Gifts program. Each segment was radiographed (Faxitron, HP, McMinnville, OR, USA) to exclude existing pathology or fractures and then was submerged in a saline bath to simulate soft tissues. Bone Mineral Density (BMD) measured for each vertebra using a DXA scanner (QDR 2000+, Hologic, Inc., Waltham, MA, USA) was performed. The donor segments were dissected clean of all musculature, with care taken to leave the posterior structures and ligaments intact, wrapped in saline soaked gauze in plastic bags and then stored in a −20° freezer.

### Magnetic resonance imaging

The spines were removed from the freezer, allowed to thaw wrapped in saline gauze at 4°C for approximately 8 h and then vacuum degassed for a period of 8 h in polyethylene tubes filled with saline at 20°C. A GE 1.5 Tesla MR scanner (General Electric Medical Systems, Waukesha, WI, USA) was used to obtain sagittal T2-weighted (TR/TE:2000/80) ms images and three sets of axial images (TR/TE: 2000/80, 2000/60, 2000/20) ms with a field of view: 150 mm and matrix size:128 × 128. The first and last axial image represented T2-weighted and proton density (PD) weighted images. A series of six phantoms with known volume fractions of heavy water (D_2_O) and saline, placed under the tubes during imaging, provided quantification of the hydration state of the disks. At the end of the imaging session, the segments were removed and returned to the freezer until the day of testing. This was necessary because of the time constraints of both the imaging and testing.

The axial T2 images were analyzed in a custom program (V5.0, Advanced Visualization Systems, Waltham, MA, USA) and the cross-sectional areas of the whole disk, the annulus and the nucleus were segmented. For each region, area and average intensity were computed and the mid-disk sagittal images used to measure the anterior, central, and posterior heights of the intervertebral disks, computed as the smallest distance between adjacent bony endplates. From the PD weighted images, relative intensity of disk areas to phantom intensity was computed and hydration level (percent H_2_O) quantified using a linear regression.

### Characterization of dynamic properties

**Figure 1:**
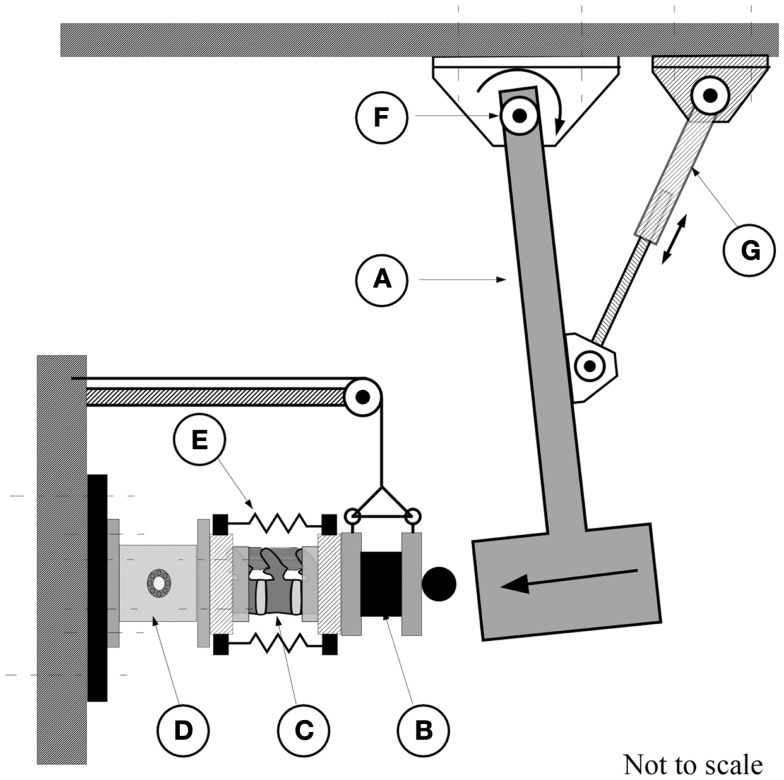
**The pendulum apparatus consisted of the pendulum arm (A) which strikes the end-mass of 20.9 kg (B) attached to the posterior caudal end of the spine segment (C)**. The cranial, potted, end of the spine was attached to a six degrees of freedom force transducer [AMTI, MC5-10000, **(D)**] which is secured to the wall. **(E)** Rubber bands for application of increasing calibrated compressive force. An electrical actuator **(G)** was used to displace the pendulum to obtain the required height for the application of the test. The arc of travel was measured by a rotary variable differential transformer [RVDT, **(F)**].

A custom impact tester (Figure 1) was used to characterize the effect of preload on the dynamic response of the FSU. This tester has been used previously by Robinovitch et al. (1997). The device consisted of a steel beam-based pendulum to which a load tray was secured allowing fine adjustment of the pendulum mass. An RVDT transducer attached at the base of the pendulum (R30A, Measurement Specialties, Shrewsbury, MA, USA), provided measurement of the pendulum arch of motion. An electrically controlled actuator, attached through a bearing to the pendulum, allowed for adjustment of the pendulum height. A 6-degree of freedom load cell (MC5-5000, resonance frequency of 1250 Hz, AMTI, Watertown, MA, USA), secured to the stationary base of the pendulum, was used to measure the transmitted force resulting from the impact (Figure 1).

Prior to testing, each FSU was thawed at 4°C for 6 h followed by a 2 h immersion in saline at room temperature under a constant compressive load of 178 N to re-hydrate the disks. This load represents the upper body mass of the lightest specimen and was chosen to avoid the potential of fracturing the more osteoporotic spines. An alignment jig was used to embed the L1 and L3 vertebrae in polymethylmethacrylate cement (Fastray, Bosworth, Skokie, IL, USA) with the specimens wrapped in plastic to prevent dehydration. The embedded spine was secured to the impact apparatus (Figure 1) and the pendulum arm, having an end mass of 20.9 kg, positioned to deliver a kinetic energy load of 2.50 J. Based on our preliminary studies, this value allowed for quantification of the dynamic response of the segment under repeated tests without causing fracture of the vertebral structures (Wilson, 1999). For all preceding tests, the force channel was sampled at a rate of 3000 Hz (V.8.0 FDS, Labview, National Instruments, TX, USA) with data acquisition triggered once the RVDT indicated that the pendulum had traveled through 5° of rotation.

The spine was first tested with no-axial preload (*P*_0_), examined for evidence of structural damage and, if none occurred, the test was repeated for the following compressive preloads; (*P*_1_): 30 ± 8 N, (*P*_2_): 79 ± 11 N, and (*P*_3_): 112 ± 14 N, respectively. To prevent the occurrence of an inadvertent rebound of the pendulum, the pendulum was restrained immediately post-impact. These preloads were applied using an increasing number of 7′′ rubber bands, with the order of preload applications randomized between specimens. This method allowed for free deformation of the spine under impact while keeping the test imposed kinematic constraints to a minimum. Using an FSU analog (made of variable rate compression springs, *k* = 362.9 ± 10.30 kN/m, *B* = 175.5 ± 14.7 Ns/m), preliminary tests found the computed error in the stiffness measurement to be <3% for the stiffness and 9% for the damping (ANOVA, *p* > 0.05). Measurements of the change in stiffness due solely to the use of the rubber bands showed no significant effect on either stiffness or damping measurements at the different preload levels.

### Data analysis

In MATLAB (V.12, MathWorks, Natick, MA, USA), fast Fourier transform (FFT) was applied to the force signal from the time where the pendulum left the end mass to obtain the peak frequency (Figure 3). A finite impulse response (FIR) low pass filter, with Hamming windowing and a cutoff frequency of twice the peak frequency, was applied to filter the signal. A damping ratio was computed by fitting a linear model to the logarithms of several successive peaks of the response. A parallel stiffness and damping model with an end mass of 20.9 kg was used to compute both dynamic stiffness (K) and damping (B) parameters. An analysis of variance (JMP 9, SAS, NC, USA) was used to test for the effect of preload (*P*_0_–*P*_3_) on the stiffness and damping constants. For each preload group, linear regression was used to test for the correlation between the dynamic stiffness and damping values and the T1 and T2 values and between these parameters and the ultimate failure strength. Statistical significance was set at 0.05.

## Results

Vertebral BMD was found to have a [mean (SD)] of 0.72 (0.19) g/cm^2^ for the L1–L3 vertebrae (Table 1), representing a range consistent with that of osteopenic vertebrae (Kanis and McCloskey, 1992). For the complete disk, both T2 intensity and relaxation values were negatively correlated with the increase in disk height (*r*^2^ = 0.35, *p* < 0.01 and *r*^2^ = 0.28, *p* < 0.05) with T2 relaxation correlated with normalized PD measures, i.e., PD/D_2_O phantom (*r*^2^ = 0.48, *p* < 0.001). In the nucleus, PD was negatively correlated with the increase in age (*p* < 0.05) while being positively correlated with T2 relaxation values (*r*^2^ = 0.52, *p* < 0.001). No such correlations were found for the annulus. No statistically significant association were found between BMD values and either the MR parameters or disk geometry (height, cross-sectional area), *p* > 0.05.

**Table 1:** **Correlation coefficients (*r*) relating stiffness constants with MRI based measurements and with age, height, and gender**.

Measured property	*M* (SD)	Correlations (*r*)			
		*P*_0_	*P*_1_	*P*_2_	*P*_3_
Preload (N)	–	0.00	−0.11	−0.32	0.19
Age	75.5 (6.9)	−0.40	−0.54*	−0.31	−0.11
Gender (0 – women)	–	0.32	0.41	0.38	0.24
Height (cm)	163 (33.0)	0.20	0.06	−0.01	0.12
Disk area (cm^2^)	16.8 (3.2)	−0.05	−0.09	0.01	0.04
Mean disk height (cm)	0.9 (0.2)	0.52*	0.46*	0.05	0.07
T2 intensity (disk)	5.6 (0.2)	−0.47*	−0.39	−0.12	0.04
T2 intensity (nucleus)	5.8 (0.3)	−0.42	−0.34	−0.31	−0.13
T2 relaxation time (disk) (ms)	65.6 (16.0)	−0.39	−0.38	−0.06	0.21
T2 relax. time (nucleus) (ms)	75.6 (24.8)	−0.38	−0.37	−0.22	0.07
T2 nucleus/annulus intensity	1.3 (0.4)	−0.20	−0.21	−0.42	−0.09
Proton-dens intensity (disk)	6.8 (0.1)	−0.17	−0.05	0.09	0.04

Gender was assigned as 0 for women and 1 for men. Intensities are relative to the heavy water phantoms to compute % of disk hydration. Values marked with a * are significant at the 5% level. Preloads groups: *P*_0_ = 0 N; *P*_1_ = 30(8) N; *P*_2_ = 79(11) N; and *P*_3_ = 112(14) N.

### Dynamic characterization

**Figure 2:**
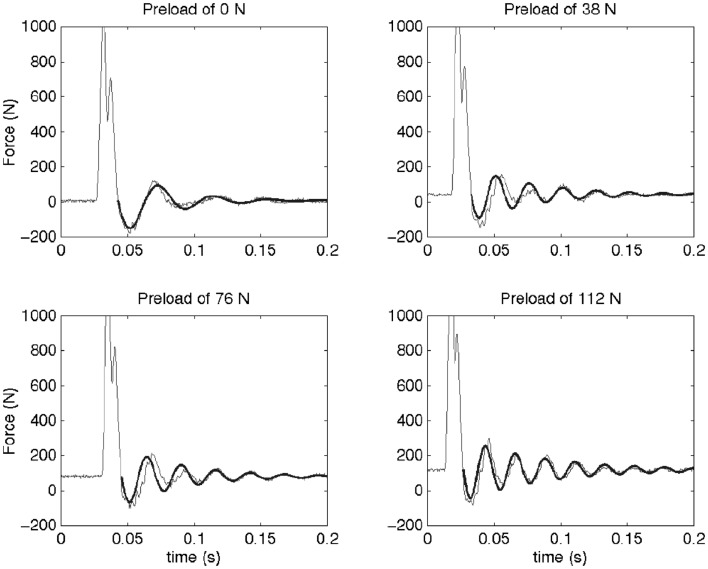
**This figure shows the force measured as a function of time for a typical sample at the four different preloads (thin line) and the fitted model (thick line)**. An initial impact is followed by a resonance of the spine segment and mass system. The dynamic behavior of a Kelvin–Voigt model was fit to the post-impact oscillations (after the force first passes across preload levels). It can be observed that the frequency of this oscillation increases with preload. In the Kelvin–Voigt model, this is represented by an increase in the stiffness of the system.

**Figure 3:**
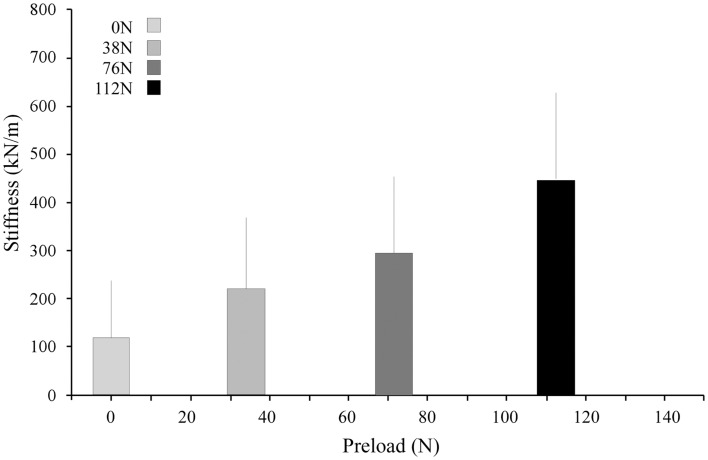
**Stiffness increased with increasing preload**. The bar graphs represent the means and standard deviations of the four preload groups.

Under applied impact, the FSU’s showed second-order, under-damped, oscillation (Figure 2). Average *r*^2^ value for a second-order fit was 0.84. Unloaded (*P*_0_), the spine exhibited a mean for dynamic stiffness of 135.3 (SD 127.6) kN/m and damping of 372.2 (SD 121.4) Ns/m. Increased preload yielded increased FSU dynamic stiffness; [*P*_1_: 210.6 (SD 164.1); *P*_2_: 293.4 (SD 157.6); and *P*_3_: 420.4 (SD 203.8)] kN/m, Figure 3. By contrast, increased preloading had little effect on the damping coefficients [(*P*_1_: 361.4 (SD 111.3); *P*_2_: 383.1 (SD 168.6); and *P*_3_: 376.7 (SD 159.9) Ns/m]. Repeated measure ANOVA demonstrated significant differences in dynamic stiffness (*p* < 0.001) between the four preload groups (*P*_0_–*P*_4_), but not for the damping coefficients.

### MR parameters vs. mechanical response

**Figure 4:**
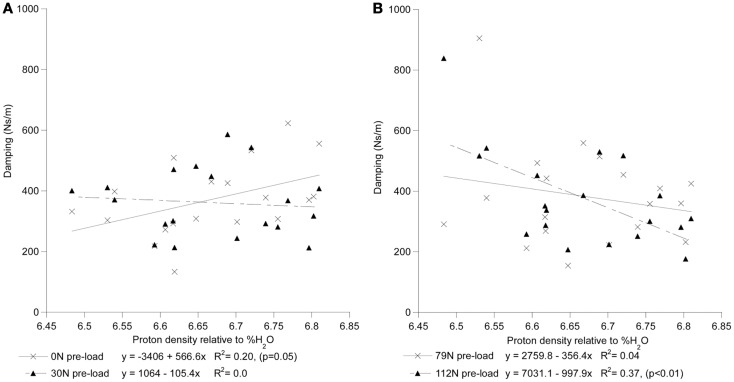
**The interrelationship of disc hydration, assessed via measurement of MR proton density, and the disc’s viscoelastic damping coefficient as a function of increased preload [(A) 0 and 30N; (B) 79 and 112N]**.

**Figure 5:**
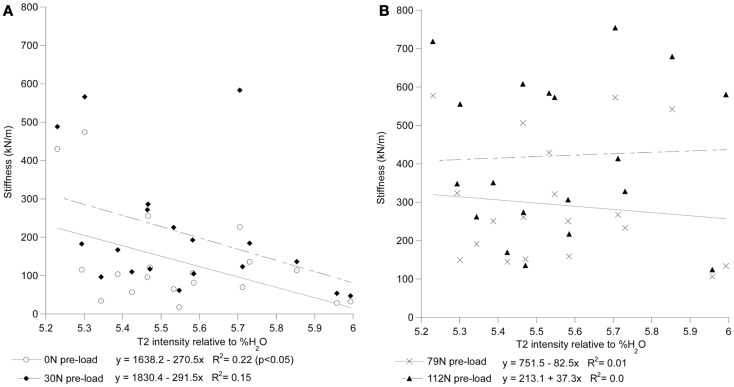
**T2 intensity of the whole disk correlates with stiffness at low preloads but not at high preloads**. In **(A)**, the stiffness of the 0 N and 30 ± 8 N groups can be seen to decrease with increased T2 intensity. In **(B)**, the stiffness of the 79 ± 11 N and 112 ± 14 N preload groups can be seen to have little correlation with T2 intensity. T2 intensity is reported relative to the scale of the heavy water phantoms where 0 is equivalent to the D_2_O phantom and 100 is equivalent to the H_2_O phantom.

For each preload group, Tables 1 and 2 presents the correlations of stiffness and damping with the MR parameters as well as age, height and gender (0 = females, 1 = males). Higher PD values were correlated with the viscous damping coefficient at 0 preload condition (*P*_0_: *r*^2^ = 0.20, *p* = 0.05, Figure 4A), but were negatively correlated with the viscous damping at the highest level of preload (*P*_3_: *r*^2^ = 0.36, *p* < 0.01, Figure 4B), Table 2. T2 relaxation and T2 image intensity were negatively associated with the dynamic stiffness (Table 1). However, this correlation was significant at low preloads (Figure 5A) and was not significant at higher preloads (Figures 5A,B).

**Table 2:** **Correlation of damping constants with MRI based measurements and with age, height, and gender**.

Measured property	Correlations (*r*)			
	*P*_0_	*P*_1_	*P*_2_	*P*_3_
Preload (N)	0.00	−0.20	−0.06	0.46
Age	−0.09	−0.04	0.40	0.37
Gender (0 – women)	0.13	0.02	−0.29	0.10
Height	−0.32	0.14	−0.29	−0.09
Disk area	0.00	0.01	0.12	0.44
Mean disk height	−0.01	−0.11	−0.40	0.17
T2 intensity (disk)	0.17	−0.16	0.12	−0.19
T2 intensity (nucleus)	0.04	−0.29	−0.02	−0.25
T2 relaxation time (disk)	−0.05	−0.04	0.17	0.10
T2 relax. time (nucleus)	−0.10	−0.19	0.12	0.01
T2 nucleus/annulus intensity	−0.15	−0.31	−0.06	−0.11
Proton-dens intensity (disk)	0.45*	−0.09	−0.20	−0.61**

Gender was assigned as 0 for women and 1 for men. Intensities are relative to the heavy water phantoms to compute% of disk hydration. Values marked with a * and **are significant at the 5 and 1% level respectively. Preloads groups: *P*_0_ = 0 N; *P*_1_ = 30(8) N; *P*_2_ = 79(11) N; and *P*_3_ = 112(14) N.

## Discussion

Developing accurate dynamic spine injury based models is critical for establishing criteria for prediction of risk of VFx in the elderly and for modeling vibration transmission due to whole body exposure. The stiffness and damping characteristics of the spine are important components in models of impact and vibrational forces on the spine. This study is the first to experimentally investigate the role of preloading on the dynamic response of the elderly intervertebral disk in combination with high-rate loading simulating a fall. With MR being the standard for diagnostic imaging of the degenerative state of the intervertebral disk, the study did support that clinical MR parameters could be used to predict some changes in the dynamic properties of the disk (particularly those at low loads).

The stiffness and damping characteristics of the intervertebral disk form a critical component in determining the spine’s dynamic response (Keller and Spengler, 1987; Shirazi-Adl, 1992; Kong and Goel, 2003; Verver et al., 2003; Van Toen et al., 2012). Our study demonstrated stiffness of the disk increases with increasing preload (Figure 5), a finding in agreement with other assessments of preload and stiffness (Rostedt et al., 1998). This suggests that the effective stiffness of a spine segment is non-linear and therefore changes with the amount of force applied. Damping, however, was found not to change with preload. Dynamic stiffness, computed in this study for the lumbar FSU from the viscoelastic model, can be compared to experiments with a single FSU by doubling the stiffness and damping values measured in this study, e.g., a single FSU would have twice the stiffness of two identical FSUs in series. Our results are in agreement with that reported for lumbar FSU under cyclic loading ranging from 17.9 to 755 kN/m (Kasra et al., 1992) and with the observed effect of preload on the stiffness (Rostedt et al., 1998). However, these stiffness values are higher than the “instantaneous” stiffness values reported for creep response of spine (Keller and Spengler, 1987) while being generally lower than those reported for axial stiffness of spine segments (Rostedt et al., 1998). Apart from the normal variation expected due to differences inherent to biological cadaveric samples, these differences may reflect both the difference in a number of factors including: (a) test conditions, (b) impact vs. cyclic or creep test, and (c) age of our sample population. The sample population in this study was chosen to allow a more accurate representation of the population that might be susceptible to osteoporotic VFx.

For models of VFx, the increase in the ratio of instantaneous (E1) to long term (E2) stiffness of the disk (Keller and Spengler, 1987) was predicted to amplify the compressive loads on the vertebrae yielding an increased risk of VFx in the event of a backwards fall from standing height (Wilson, 1999). The model further predicted that a one standard deviation (163 kN/m) increase in stiffness will result in changes in the predicted forces on the spine during a backwards fall by as much as 18%. Such an increase, for example, would cause the predicted axial force of the one impact model to increase from 2505 to 2948 N. With elderly cadaveric vertebrae reported to have an average axial failure force of approximately 2000 N (Moro et al., 1995), this predicted increase may become highly significant in affecting the onset of VFx.

By contrast, higher viscoelastic damping coefficient of the disks demonstrated a stress dependent association with the increase in the estimated hydration of the disk (Figure 5). These findings, in agreement with the reported effects of strain (Lee and Teo, 2004) and loading frequency (Iatridis et al., 1997; Kuo and Wang, 2010) on the permeability of the disk tissues, suggest disk hydration plays a significant role in the immediate response of the spine to sudden impact. Under impact loading, our computational model predicted the immediate (up to 200 ms) response of the FSU resulting in a constant pore pressure within the nucleus, rather than the solid matrix of the disk, supports the compressive load for the duration of the impact (Lee et al., 2000). Furthermore, with the annulus restricting the motion of fluid normal to the axial plane of the disk, the model predicted that high fluid flow through the endplates to result in the development of high pressure within the vertebral cancelous bone, contributing to and/or resulting in endplate and vertebral cortex fractures. The findings of this study provide, for the first time, a possible experimental validation of computational model predictions on the role nucleus hydration status plays in the ability of the spine segment to support high-rate loading. Though it is clear that both the application of preloading (Wang et al., 1999; van Engelen et al., 2011) and the relative contribution of spinal osseoligamentous tissues form and important contribution to the static (Brown et al., 1957; Adams and Hutton, 1983; Adams et al., 1996; Van Toen et al., 2012) and dynamic response (Kasra et al., 1982; Kemper et al., 2007) of the spine, the findings of this study strongly suggest that future assessments of the force propagation in the spine should take into account this non-linear behavior in predicting the fracture risk of the spines under impact loading.

Increased disk degeneration, underlined by the biological (Bibby et al., 2001) and physiological (Acaroglu et al., 1995) modification of the disk’s tissues, results in the significant degradation of its static, dynamic, and viscoelastic properties (Kasra et al., 1982; Burns et al., 1984; Keller and Spengler, 1987; Best et al., 1994; Iatridis et al., 1997, 1998; Bibby et al., 2001; Pollintine et al., 2010; O’Connell et al., 2011). Magnetic resonance based assessment of disk degeneration and loss of water content (including decreased T2 relaxation time and T2-weighted image intensities) were found to correlate significantly with an increase in stiffness of the disk only at low preloads (Table 2). The observed lack of correlation could have been the result of the MRI measurements being done with no preload applied which is known to effect dynamic response of the FSU (Wang et al., 1999; van Engelen et al., 2011). However, this scanning regime simulated a clinical MRI assessment typically done in a prone posture during which the compressive load on the spine is likely low. In agreement with the study by Chiu et al. (2001), the results of this study suggest that current clinical MR based classification of degenerative changes may not reflect the upright, loaded characteristics of the spine and thus should be used with caution to directly assess the loss of dynamic stiffness and damping properties of the spinal segments.

This study suffers from the obvious limitations faced when attempting to translate an *in vitro* study to the clinical setting. Although Panjabi et al. (1985) found that biomechanical properties of the spine did not change with freezing, the specimens in this study were forced to go through several freezing and thawing cycles, including one between imaging and testing which may have had some effect on the tissue properties. This study was also performed at room temperature rather than at body temperature. Little is known about the effects of temperature on the viscoelastic behavior of the intervertebral joint, but it is possible that temperature could play a significant role. Though we have attempted to standardize the testing methodology, little knowledge exists with respect to the loading conditions which occur on the spine due to a fall event, or, the contribution of the muscular system to the stability and load attenuation of the spine. During a fall, it is likely that both the geometrical and kinematic configuration of the spinal column will vary considerably from normal axial alignment resulting in a complex set of loading modes (Wilson, 1999). Axial compression mode testing was used as it represents the simplest mode of loading which may occur on the spine due to a fall event. The Kelvin–Voigt model of the FSU dynamics is often used in lumped parameter models of vibration transmission and spine dynamics as it is reasonably straightforward to implement and represents the viscoelastic dynamics well. However, the changes in stiffness with preload observed in this experiment demonstrate that the linear Kelvin–Voigt model, or even a linear Kelvin–Voigt–Maxwell model, may not capture the full viscoelastic dynamics of the spine due to the linear nature of the stiffness components. A non-linear spine model may be necessary for better accuracy.

In conclusion, stiffness during impact loading of L1–L3 spine segments was found to range from 17.9 to 755 kN/m and was significantly related to the preload applied. Models existing in the literature currently model the stiffness as a single, linear value. However, a non-linear stiffness component may be necessary to more accurately describe models of impact due to a backward fall in order to reflect the changes of the stiffness at different force levels as seen in this experiment. Degenerative changes in intervertebral disks, measured by decreases in MRI by T2 relaxation time and T2-weighted images, were found to be weakly correlated with stiffness. MR measurement of disk hydration were significantly correlated with the viscoelastic and ultimate strength of the FSU’s, suggesting fluid flow mechanism within the disk and vertebrae to be an important determinant of the ability of the spine to sustain high-rate loading.

## Conflict of Interest Statement

The authors declare that the research was conducted in the absence of any commercial or financial relationships that could be construed as a potential conflict of interest.
